# Cellulose Nanocrystals and Lignin Nanoparticles Extraction from *Lemna minor* L.: Acid Hydrolysis of Bleached and Ionic Liquid-Treated Biomass

**DOI:** 10.3390/polym16101395

**Published:** 2024-05-14

**Authors:** Debora Puglia, Francesca Luzi, Ciro Tolisano, Marco Rallini, Dario Priolo, Monica Brienza, Ferdinando Costantino, Luigi Torre, Daniele Del Buono

**Affiliations:** 1 Department of Civil and Environmental Engineering, University of Perugia, UdR INSTM, 05100 Terni, Italy; marco.rallini@unipg.it (M.R.); luigi.torre@unipg.it (L.T.); 2 Department of Science and Engineering of Matter, Environment and Urban Planning (SIMAU), Polytechnic University of Marche, UdR INSTM, 60131 Ancona, Italy; f.luzi@staff.univpm.it; 3 Department of Agricultural, Food and Environmental Sciences, University of Perugia, 06121 Perugia, Italy; ciro.tolisano@studenti.unipg.it (C.T.); dario.priolo@unipg.it (D.P.); daniele.delbuono@unipg.it (D.D.B.); 4 Dipartimento di Scienze, Università degli Studi della Basilicata, Via dell’Ateneo Lucano 10, 85100 Potenza, Italy; monica.brienza@unibas.it; 5 Dipartimento di Chimica, Biologia e Biotecnologia, University of Perugia, Via Elce di Sotto 8, 06123 Perugia, Italy; ferdinando.costantino@unipg.it

**Keywords:** duckweed, aquatic plant, cellulose nanocrystals, lignin nanoparticles, sulfuric acid, ionic liquid

## Abstract

Using biomass to develop and obtain environmentally friendly and industrially applicable biomaterials is increasingly attracting global interest. Herein, cellulose nanocrystals (CNCs) and lignin nanoparticles (LNPs) were extracted from *Lemna minor* L., a freshwater free-floating aquatic species commonly called duckweed. To obtain CNCs and LNPs, two different procedures and biomass treatment processes based on bleaching or on the use of an ionic liquid composed of triethylammonium and sulfuric acid ([TEA][HSO_4_]), followed by acid hydrolysis, were carried out. Then, the effects of these treatments in terms of the thermal, morphological, and chemical properties of the CNCs and LNPs were assessed. The resulting nanostructured materials were characterized by using Fourier-transform infrared (FTIR) spectroscopy, X-ray diffraction (XRD) spectroscopy, thermo-gravimetric analysis (TGA), and scanning electron microscopy (SEM). The results showed that the two methodologies applied resulted in both CNCs and LNPs. However, the bleaching-based treatment produced CNCs with a rod-like shape, length of 100–300 nm and width in the range of 10–30 nm, and higher purity than those obtained with ILs that were spherical in shape. In contrast, regarding lignin, IL made it possible to obtain spherical nanoparticles, as in the case of the other treatment, but they were characterized by higher purity and thermal stability. In conclusion, this research highlights the possibility of obtaining nanostructured biopolymers from an invasive aquatic species that is largely available in nature and how it is possible, by modifying experimental procedures, to obtain nanomaterials with different morphological, purity, and thermal resistance characteristics.

## 1. Introduction

*Lemna minor*, commonly known as duckweed, is a small, free-floating aquatic species that belongs to the *Lemnaceae* family. Although the composition of duckweed can vary slightly depending on factors such as environmental conditions, growth stage, and geographical location, this species contains, in addition to water, which makes up a considerable portion of its fresh weight, carbohydrates, including cellulose, hemicellulose, and starch, which serve as structural components and energy reserves [1]. The literature on the chemical composition of plants appertaining to the *Lemnaceae* family reports that the content of polysaccharides (starch, cellulose, hemicellulose, and pectin) is 17.6–35%, lipids is between 3.4 and 9.0%, minerals is 3.5–26%, and proteins is 16–41.7%, along with various phytochemicals (phenols, glucosinolates, lutein, β-carotene, α-tocophenols, and others) [2]. Specifically, duckweed contains protein (26.8%), soluble sugars (51.1 ± 1.0%), and total amino acids (9.0 ± 5.2%) on a fresh weight basis [3]. Furthermore, this plant has a significant content of fibers (48.9%), with lignin as the phenolic-based bio-polymeric macromolecule responsible, among other structural and protective functions, for polysaccharide binding, which is found in a proportion ranging from 5–12.2% of the cell wall, depending on the plant’s vegetative stage and growth conditions [4,5]. In addition, duckweed contains a significant amount of molecules and hormones essential for growth, development, and various metabolic processes, as well as a plethora of bioactive compounds [1,6]. In fact, duckweed may contain vitamins and various compounds, including antioxidants and flavonoids, which contribute to its health benefits and defense mechanisms against environmental stresses [7].

Duckweed has been studied for its potential applications in various fields, including hydrogels with topic antibacterial functions [8], crop protection [9], wastewater treatment [10], biofuel production [11], nutrient provision for humans and animals [6,7], and for biogas production [4,12,13]. In addition, this species can absorb many different substances from its aquatic environment, including essential nutrients, such as nitrogen, phosphorus, potassium, calcium, magnesium, and also toxic elements [14]. The contents of cellulose, hemicelluloses, lignin, and available proteins can play a vital role in the plant’s capacity to absorb metal trace elements (MTEs) and other water pollutants [15,16]. It has been demonstrated that aquatic plants can efficiently remove MTEs from aqueous solutions [17]. Furthermore, when extracted from plants, lignocellulosic materials have proven to be effective MTE adsorbents, and the use of cellulose and lignin obtained from waste as potential low-cost biosorbents has been explored as a particularly attractive option [18,19]. Despite this, for aquatic species, over the last decade, few studies have paid attention to them, focusing on the isolation of nanocellulose (including nanocrystals and nanofibers) by acid hydrolysis, as in the case of water hyacinth [20,21,22], while no examples of extracted nanosized lignin can be found for these plants. It should also be underlined that chemical pretreatments of these species, particularly ones that use NaOH and NaClO_2_ in acidic conditions (for bleaching or delignification), are some of the most common procedures that aim to isolate cellulose with, on the other hand, low in-process lignin residue generation [23]. Most available studies did not address this point, and lignin recovery was mainly not attempted. According to this, a sustainable and effective pretreatment method should be found, aiming to obtain both lignin and cellulose from aquatic plants.

Ionic liquids based on hydrogen sulfate anions obtained by a reaction between an organic Lewis base and mainly sulfuric acid are suitable for this purpose [24]. The physicochemical characteristics of ILs allow them to diffuse effectively into the fibers, particularly the cellulose chains, and disaggregate and separate them. The reactive nucleophilic oxygen in the hydrogen sulfate and the possibility of this group and the Lewis base forming strong hydrogen bonds result in a particular efficacy of ILs in breaking covalent bonds and robust non-covalent interactions among fiber components [25]. Such an effect leads to the breakdown of the fibers, thus allowing for the separation and recovery of cellulose and lignin [25]. In addition, depending on temperature, precipitation time, pH, and other factors, it is possible to generate the latter biopolymer in nanoscale [26].

In this context, the present study aimed to obtain cellulose and lignin nanoparticles from duckweed, ascertaining the effects on the final results of two pretreatment processes (bleaching and triethylammonium sulfate [TEA][HSO_4_] ionic liquid treatment, followed by sulfuric acid hydrolysis). Finally, the obtained nanosized cellulose and lignin’s thermal, morphological, and chemical properties were assessed.

## 2. Materials and Methods

### 2.1. Materials

Duckweed was harvested from a freshwater basin near Perugia (Italy). Initially, the plants underwent sterilization with a 0.5% sodium hypochlorite solution for 2 min. Then, the plants were copiously rinsed twice with distilled water. Subsequently, the duckweed plants were transferred to polyethylene trays (35 × 28 × 14 cm) and cultivated following a previously published protocol [27]. The culture media was replaced every two weeks. The ionic liquid (IL)—triethylammonium sulfate [TEA][HSO_4_] [stoichiometric ratio 1:1]—was synthesized by using 0.1 mol of triethylamine (TEA) placed in a 250-mL round-bottomed flask and maintained under stirring at 60 °C. After that, 0.1 mol of H_2_SO_4_ was added dropwise at 60 °C to allow the reaction with TEA, and then the mixture was left to react for 1.5 h at 70 °C. All the reagents were supplied by Sigma Aldrich (St. Louis, MO, USA).

### 2.2. Extraction of Cellulose Nanocrystals (CNC)

#### 2.2.1. Bleaching Treatment

The duckweed plant was washed 3 times in acetone (1:20 *w*/*w*) to remove interfering substances, and then the material was dried at RH for 24 h. The green color of the acetone was because it acted as a detergent/solvent, breaking down the phospholipid bilayer and opening holes in the membrane, making it permeable and promoting the elimination of the chloroplasts [28]. In order to obtain complete whitening of the fibers, two bleaching treatments were applied for cellulose extraction: first, for 2 h with a 0.7% (*w*/*v*) solution of sodium chlorite NaClO_2_ and fibers (1:50 *w*/*v* ratio). The pH of the solution was lowered to approximately 4 with acetic acid. After that, 5% (*w*/*v*) sodium bisulfate solution was applied. Finally, the holocellulose was treated with a 17.5% (*w*/*v*) NaOH solution to obtain α- cellulose. The fibers were washed thoroughly with deionized water and dried.

#### 2.2.2. Acid Hydrolysis after Bleaching Treatment

**Scheme 1:**
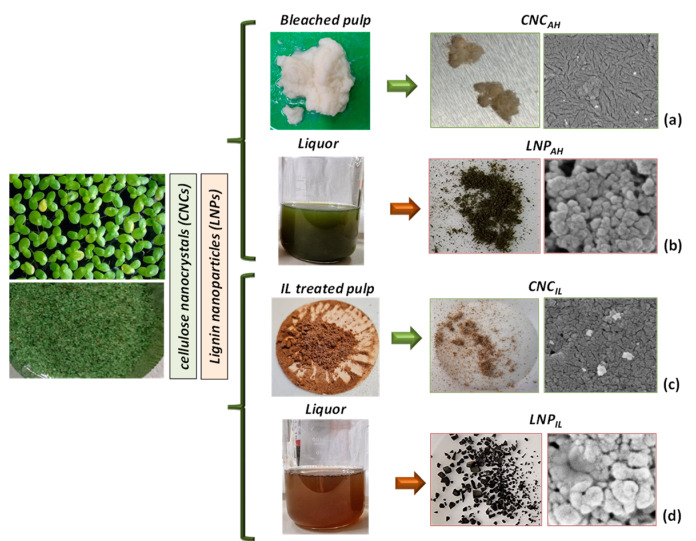
Images of liquor, pulps, and CNC (**a**,**c**) and LNP (**b**,**d**) micrographs after IL and bleaching treatments.

Cellulose nanocrystal (CNC_AH_) suspensions were prepared from chemically pre-treated duckweed, as described in Section 2.2, by sulfuric acid hydrolysis (reagent 98%) [29,30]. Hydrolysis was carried out with 64% *w*/*w* sulfuric acid at 45 °C for 30 min with vigorous stirring. Immediately after acid hydrolysis, the suspension was diluted 20 times with deionized water to quench the reaction. The suspension was centrifuged at 4200 rpm for 20 min to concentrate the cellulose crystals and to remove the excess of aqueous acid. The resultant precipitate was rinsed, re-centrifuged, and dialyzed against deionized water for 5 days until a constant neutral pH was achieved. The suspension was sonicated repeatedly at 40% output (while cooled in an ice bath) to create cellulose crystals of colloidal dimensions (Scheme 1a).

#### 2.2.3. Acid Hydrolysis after Ionic Liquid Treatment

Cellulose nanocrystal (CNC_IL_) suspensions were prepared by acid hydrolysis of the residual pulp fraction after IL treatment for lignin separation (as described in the following Section 2.3) (Scheme 1c). Hydrolysis was carried out with 64% *w/w* sulfuric acid at 45 °C for 30 min with vigorous stirring. Immediately after acid hydrolysis, the suspension was diluted 20 times with deionized water to quench the reaction. The suspension was centrifuged at 4200 rpm for 20 min to concentrate the cellulose crystals and to remove the excess aqueous acid. The resultant precipitate was rinsed, re-centrifuged, and dialyzed against deionized water for 5 days until a constant neutral pH was achieved. The suspension was sonicated repeatedly at 40% output (while cooling in an ice bath) to create cellulose crystals of colloidal dimensions. (Scheme 1c).

### 2.3. Lignin Nanoparticles Extraction (LNP)

#### 2.3.1. Lignin Precipitation after Bleaching Treatment

Five grams of dried duckweed plant sample were added to a NaOH solution (17.5% *w/v*) in a three-necked round bottom flask. The treatment process was operated at 100 °C for 90 min. The treated solution was filtered through Whatman filter paper to remove the solid component. During the second step, the pH of the liquid solution was modified by adding HCl and, consequently, the lignin started to precipitate. The acid solution was maintained for 12 h until the top layer of the solution became transparent. The bottom layer of the liquid containing the suspension of lignin particles (LNP_AH_) was centrifuged for 20 min at 4200 rpm (Scheme 1b). The obtained LNP_AH_ was washed 3 times to remove and dissolve the salt produced during the neutralization reaction. The purified solution was lyophilized [31].

#### 2.3.2. Lignin Precipitation after IL Treatment

Two grams of dry duckweed biomass were mixed with [TEA][HSO_4_] (1:20, *w*:*w*), containing 10% wt. of water (90% IL—10% water). The mixture was then left at 120 °C for 4 h. Once the extraction was completed, the mixture was left to cool at room temperature, and 54 mL of ethanol (EtOH) was added to the mixture, which was filtered to eliminate the solid residue. The EtOH was then evaporated, and 170 mL of H_2_O was added to precipitate the lignin. Finally, the lignin suspension (LNP_IL_) was centrifuged and washed more times with water, then with 1% formic acid, and left to dry at 60 °C in the oven (Scheme 1d).

### 2.4. Characterization of Pristine Duckweed Plant, Cellulose Nanocrystals and Lignin Nanoparticles

The microstructures of the pristine duckweed plant and CNC and LNP powders were investigated by means of field emission scanning electron microscopy using a FESEM Supra 25 (Zeiss, Oberkochen, Germany). Powders of CNC and LNP were swollen in distilled water before FESEM observation. A 1% wt. aqueous solution was stirred for 4 h at room temperature. The solutions were then subjected to 1 h of sonication in 10 min intervals in order to loosen up the particles. A few drops of the CNC and LNP suspensions were cast onto a silicon substrate, vacuum-dried, and gold-sputtered before the analysis.

Fourier-transform infrared (FT-IR) spectra of the pristine duckweed plant, cellulose nanocrystals, and lignin nanoparticles (CNC_AH_, CNC_IL_, LNP_AH_, and LNP_IL_) were recorded in the transmission mode using KBr discs.

Thermogravimetric measurements (TGA) of pristine and extracted materials (CNC_AH_, CNC_IL_, LNP_AH_, and LNP_IL_) were performed by using a Seiko Exstar 6300 analyzer (Tokyo, Japan) in order to evaluate the effect of pretreatments and extraction procedures on the thermal behavior of the different samples. Heating scans from 30 to 900 °C at 10 °C min^−1^ under a nitrogen atmosphere were performed for each sample.

XRD analysis was performed by using Cu K radiation to examine the sample with the following working lamp parameters: V = 40 kV, I = 40 mA, and receiving slit = 0.15 mm. With a scan range of 10–50 and a scan speed of 2°*/*min, the intensity of the reflections was measured.

## 3. Results

### 3.1. Characterization of Pristine Duckweed

**Figure 1:**
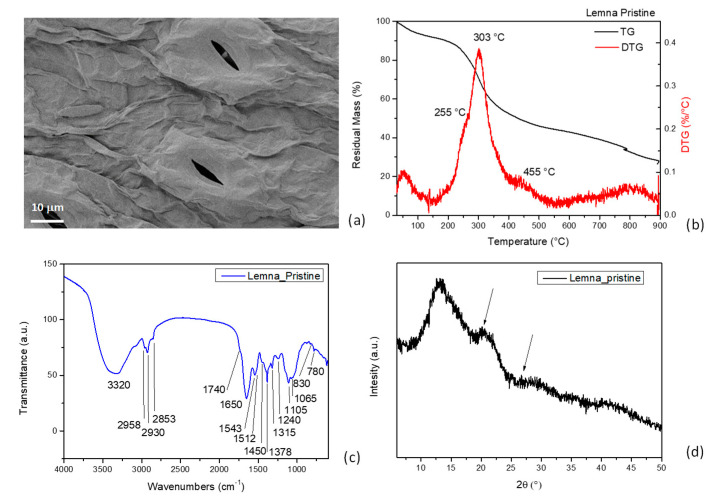
FESEM image (**a**), TG*/*DTG curves (**b**), and FTIR (**c**) and XRD (**d**) spectra of pristine Lemna.

Morphology: FESEM analysis indicated the typical anatomical structure and surface of plant fronds that was smooth and intact, with the cells well-arranged and close-spaced, and the presence of diatoms and open stomata on the dorsal side of the frond (Figure 1a) [32,33,34,35,36,37]. From the perspective of cellulose extraction, Lemnaceae produce and accumulate substantial amounts of wall mass, which can be used to obtain biofuels. For such richness and structural complexity, it is expected that even nanocellulose*/*nanolignin extraction is a challenge for this plant [36].

TGA: The analysis indicated weight loss between 100 °C and 150 °C, which can be associated with biomass dehydration. The first stage of thermal decomposition of the duckweed samples occurred in the temperature range between 200 °C and 300 °C, with maximum weight loss occurring at 270 °C, which may be attributed to hemicellulose degradation and volatilization. The main cellulose decomposition peak, located in the temperature range of 300–400 °C and with a maximum at 345 °C, overlapped with that of hemicellulose. A long tail of devolatilization was observed in the duckweed at 455 °C due to lignin loss (Figure 1b). The lower thermal stability of hemicellulose is due to its lesser organized structure compared with cellulose for the presence of randomly amorphous linked saccharides rich in branches (xylose, mannose, glucose, galactose, etc.). They can easily be degraded to volatiles such as CO, CO_2_, and hydrogen carbon compounds. Furthermore, lignin decomposes in a broader temperature range than hemicelluloses and cellulose in the 200–600 °C range, with a typical signal at around 455 °C [38]. This is due to its structural complexity and functional groups of lignin, as it is a phenols-based polymer, thus showing very different thermal stabilities [20]. On a quantitative basis, more of the volatile matters were released in the primary devolatilization than in the secondary volatilization step, as it accounted for more than 50% of the final volatile matters released at 500 °C [4].

FTIR: In the FTIR spectrum of lignocellulosic duckweed (Figure 1c), different bands for O–H stretching of polymeric compounds and polysaccharides, COO–, C–O stretching of carboxylic acids, and carbonyl group and -CH_2_ symmetric vibration are visible [39]. The broad, intense adsorption peak around 3300 cm^−1^ is indicative of the adsorption of water molecules and of the -OH hydroxyl groups and -OH stretching of phenolic groups, while other bands characteristic of C-H, C-O deformation, and bending or stretching vibrations of many groups in lignin and carbohydrates were detected. In detail, the narrow bands positioned at 2930 cm^−1^ and 2850 cm^−1^ were due to the -CH stretching of methyl and methylene in aliphatic groups and that of methoxyl in the aromatic ones [26]. The band in the 1550–1650 cm^−1^ range can be assigned to the amide bond stretching of cell wall proteins, glycoproteins, enzymes, and structural polypeptides [40]. In addition, the presence of water is also associated with the signal in this region and the presence of no esterified pectin groups [40]. The band at 1543 cm^−1^ was ascribable to the stretching vibration of C–N of the peptidic bond of proteins; 1450 cm^−1^ to phenolic -O–H and C–O stretching of carboxylates; 1315 cm^−1^ to C–H vibration in cellulose; 1240 cm^−1^ to the vibration of carboxylic acids; and 1065 and 890 cm^−1^ to the vibration of C–O–C of polysaccharides. The hemicellulose peaks are located at 1378 cm^−1^ and 780 cm^−1^, assigned to the carboxyl groups (β-glycosidic linkages (1→4) between xylose units), while the peak for amorphous cellulose molecules can be found at 830 cm^−1^ [32,41].

XRD*:* The results of XRD characterization showed that pristine duckweed exhibited diffraction peaks at 2θ around 13.0° and 20.0°, corresponding to (1 1 0), (1 1 0), typical of the cellulose structure and starch component [39,42,43] (Figure 1d). Ferrer et al. [44] reported that duckweed has a rather low crystallinity; drying also induces morphological changes in the material with high water content, modifying the crystalline structure, thus causing the change from cellulose I to cellulose II.

### 3.2. Thermal, Morphological, and Chemical Analysis of Cellulose Nanocrystals

#### 3.2.1. Acid Hydrolysis (CNC_AH_)

**Figure 2:**
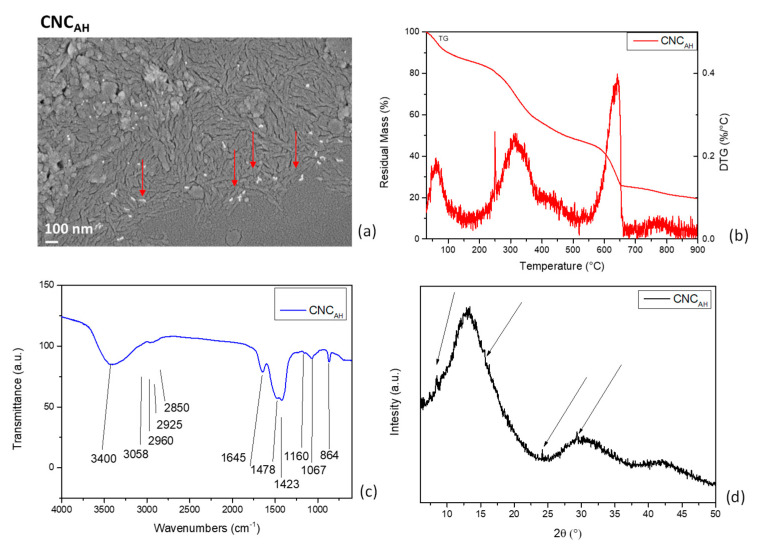
FESEM image (**a**), TG*/*DTG curves (**b**), and FTIR (**c**) and XRD (**d**) spectra of CNC_AH_. Red arrows indicate calcium oxalate crystals.

Morphology: The morphological analysis of CNC_AH_ by FESEM (Figure 2a) evidenced a rod-like structure, with a length in the range of 100–300 nm and a width of 10–30 nm. This result was due to the treatments. In particular, bleaching, which first hydrolyzed hemicellulose and removed lignin, followed by acid hydrolysis destroyed the amorphous domains of cellulosic microfibrils while retaining the straight crystalline ones [22]. The investigation also proved the presence of other nanometric structures, which were supposed to be calcium oxalate crystals. Duckweed produces significant amounts of this compound, which is mainly used in plant defensive responses [45]. These oxalate crystals can have different shapes and sizes, such as raphides and druses, the most common types found in duckweed. They are formed when calcium is taken and deposited in specialized idioblast cells [45].

TGA: The first degradation step was due to the evaporation of the residual water present in cellulose. After that, the TG*/*DTG curves (Figure 2b) of CNC_AH_ showed that cellulosic material decomposed between 300 and 400 °C owing to the decarboxylation, depolymerization, and decomposition of glycosidic linkages, confirming the disappearance of peaks due to hemicellulose and lignin, as observed in the pristine lemma. The shift of the main peak toward lower temperatures also confirmed that the extracted CNC had lower thermal stability than the pristine plant. This effect was reasonably due to the negatively charged sulfonic groups that formed on the exterior surface of the cellulose crystals for the treatments [46]. Up to 500 °C, the residual mass for CNC could also be considered higher than that of the corresponding plant, as already observed for other cellulosic nanostructures [47]. However, in accordance with what was already commented on in the morphological characterization, the thermal degradation of the extracted CNC also proved the presence of a further component degrading at upper temperatures, specifically in the range of 550–700 °C. This degradation pattern can be assigned to the decomposition of calcium (CaC_2_O_4_) or sodium (Na_2_C_2_O_4_) oxalates [48,49].

FTIR: The analysis of the obtained spectrum (Figure 2c) evidenced that the peak located at 1240 cm^−1^, attributable to ester, ether, or phenol compounds; the peak at 1740 cm^−1^, assigned to either the acetyl and uronic ester groups of hemicelluloses or the ester linkage of the carboxylic groups of lignin; and that at 1512 cm^−1^, corresponding to C=C stretching in the aromatic rings of the lignin structure; disappeared. These changes and the disappearance of the bands confirmed the removal of hemicellulose and lignin and the incremental increase in the proportion of cellulose [41,50]. The band at 3400 cm^−1^ is attributable to the hydroxyl groups of cellulose and that at 2850–2960 cm^−1^ to the stretching and deformation of the -C-H group of glucose units [21]. The deformation and vibration of the C–C bond in the ring can assigned to the peak at 1160 cm^−1^; the peak at 1067 cm^−1^ is attributable to the C–O stretching of the cellulose, and it was more intense in the acid-hydrolyzed CNC_AH_. -CH_2_ scissoring (bending) deformations were principally responsible for the bands at 1478 cm^−1^ and 1423 cm^−1^. These bands reveal details on cellulose’s intramolecular and intermolecular bonds’ ultrastructures [30]. The spectrum’s signal at 864 cm^−1^ was linked to the CH deformation of cellulose, and this peak was more noticeable after bleaching and acid hydrolysis, indicating that the cellulose content increased due to further extraction steps. The intense signal at 1645 cm^−1^ and minor one at 1316 cm^−1^ were due to the symmetrical stretching vibrations of the COO- groups, which can be attributed to oxalates [51].

XRD: The diffraction peak for pristine duckweed was relatively broader and became sharper and narrower after chemical treatments: bleaching and acid hydrolysis effectively removed the amorphous domains in the raw duckweed [50,52]. XRD characterization of cellulose nanocrystals isolated by acid hydrolysis confirmed the presence of a main peak at 13° and the absence of the one at 20°, which was present in the pristine duckweed and attributed to starch. The characteristic cellulose peak at 34–35° in the pristine duckweed, related to the crystallographic plane (004) perpendicular to the chain, was weak and hardly detectable in Figure 1d: this limited evidence can be explained by considering that aquatic floating plants have specific adaptations to their unique habitat, so changes in cell wall composition and alterations in cellulose crystallinity pattern are expected when compared with other plants. The three dominant peaks at 13°, 30°, and 42° can be associated with the crystalline structure of cellulose type II (polymorphic transition from cellulose type I to II in acid-treated cellulose) (Figure 2d) [53]. The signals of calcium oxalate could also be detected in the CNC_AH_ spectrum [54], specifically at 8.3°, 24.2°, and 29°, characteristic of this material [55].

#### 3.2.2. Ionic Liquid (CNC_IL_)

ILs are widely recognized for their capacity to form intense charged and H-bonding-based interactions, which could disturb the bonding network of plant fibers and facilitate their separation. Although most IL pretreatments do not require water or just a limited amount to preserve their effectiveness in solubilizing lignin and hemicellulose, some [HSO_4_]-based ILs have been recently reported to be effective even by adding water [56]. The addition of water to the IL [TEA][HSO_4_] (90% IL—10% water) was performed considering that the performance of this IL is positively affected by water, owing to its specific involvement in the hydrolytic processes that de-structure fibers [56]. Indeed, the mechanism of action of IL [TEA][HSO_4_] in biomass pretreatment can be seen as the breaking down of cellulose’s and hemicellulose’s hydrogen and covalent bonds with lignin, both intra- and intermolecularly [56]. In particular, the hydrogen sulfate anion is very effective in fiber decomposition, thereby reducing the recalcitrant nature of the biomass to fiber separation. Cellulose, which regenerates after IL treatment, tends to be more amorphous in its macrostructure, making it easier for it to undergo further hydrolysis [57]. Based on this assumption, the pulps originating from the [TEA][HSO_4_] treatment of pristine duckweed (see Section 3.3.2) were subjected to sulfuric acid hydrolysis for further recovery of cellulose nanocrystals (CNC_IL_) [58,59].

**Figure 3:**
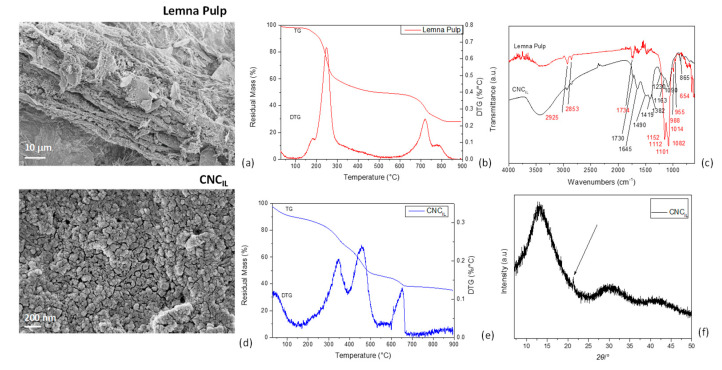
FESEM images of Lemna pulp (**a**) and CNC_IL_ (**d**), TG*/*DTG curves for lemna pulp (**b**) and CNC_IL_ (**e**), FTIR spectra of lemna pulp and CNC_IL_ (**c**), and XRD (**f**) spectrum of CNC_IL_.

Morphology: The fibrillar characteristics of cellulose pulp after IL treatment can be observed in Figure 3a. The CNCs obtained via this method were essentially spherical (Figure 3d) instead of whisker-like, obtainable by the procedure we applied or other conventional methods. Al Hakkak et al. [60] reported that cellulose aggregates in spherical nanoparticles with diameters less than 100 nm. Nonetheless, the original organization of this macromolecule in microfibrils in the pulp was disrupted, as evidenced by the acid hydrolysis, which allowed for nanosized particles to be obtained [61,62].

TGA: The thermal degradation pattern for pulp derived from duckweed after IL treatment is shown in Figure 3b. The cellulose pulp exhibited a two-step weight-loss degradation profile alongside the initial loss allocated to the release of adsorbed water below 100 °C. The first degradation stage occurred with a maximum decomposition temperature of 183 °C, while the second with a maximum decomposition temperature of 250 °C. The first degradation step can be related to the thermal decomposition of calcium oxalate [48], whereas the second main peak was linked to the decomposition of the cellulose. These results clearly prove that the pulp recovered after IL treatment had lower thermal stability than the non-modified cellulose [63], as extensively documented by the literature on the thermal behavior of IL-regenerated celluloses [64]. The weight loss between 300 °C and 500 °C, appearing as a broader peak, could be mainly related to lignin component degradation and calcium oxalate. Finally, the two signals observed in the 750–900 °C range could be assigned to the endogenous calcium and sodium oxalate, respectively [48,65]. As already observed for CNC_AH_ (Figure 2b), the CNC obtained by the acid hydrolysis of IL-treated pulp showed the typical degradative pattern of the cellulose component, with the related peak centered at 348 °C, and the additional signal of residual lignin (peak centered at 460 °C), already confirmed by the chemical analysis (Figure 3e). The TG*/*DTG profiles confirmed that IL treatment was less effective in recovering cellulose from the starting material than the other procedure, and this was due, among other reasons, to the lignin residual amounts found.

FTIR: The IR spectrum of duckweed pulp from IL treatment is presented in Figure 3c. The spectra displayed common characteristics of cellulose: the band centered at 3348 cm^−1^ was assigned to the hydroxyl group (O-H), and the peaks at 2925 and 2853 cm^−1^ indicated the C-H stretching in -CH_2_ group. The peak at 1151 cm^−1^ corresponded to the C–OH and C–O–C pyranose ring stretching vibration in cellulose. The C–C ring stretching band in cellulose could be seen in the peak at 1112 cm^−1^, indicating the crystalline cellulose I form. The peak at 1082 cm^−1^ was related to the C–O–C binding group, while the peak at 1734 cm^−1^ may be attributed to the yellow color of cellulose pulp. The absorption peak at 1101 cm^−1^ was also the stretching vibration peak of the alicyclic ether C-O-C and that at 1014 cm^−1^ was the -CH_2_ bending of cellulose. The peak at 988 cm^−1^ was attributed to cellulose C–O and C–C stretching [66], while the sharp peak at 654 cm^−1^ could be related to the -C–O-H out-of-plane bending of cellulose. Signals related to the presence of residual [TEA][HSO_4_] and lignin could be found in the range 1600–1500 cm^−1^, as lignin contains a large number of aromatic rings, and the C=C stretching vibrations of these rings appeared in this region [57,67].

The extracted cellulose was then subjected to acid hydrolysis to produce CNC_IL_ nanoparticles. The analysis of the CNC_IL_ spectrum (Figure 3c) confirmed the presence of characteristic peaks for cellulose: hydrogen-bound -OH stretching vibration at 3300–3400 cm^−1^ and peaks at 2900 cm^−1^ associated with C-H stretching vibrations. The broad absorption peak at 3350 cm^−1^ was caused by the stretching vibration of hydroxyl (–OH), 1645 cm^−1^ (HOH) was caused by adsorbed water (water absorption due to the sulfate groups of sulfuric acid becoming placed on the surface after the hydrolysis process), and the band at 1730 cm^−1^ corresponded to the C–O–O stretching vibration. The signal at 1163 cm^−1^ (C-O-C) was related to the ring glycosidic link, while that at 1419 cm^−1^ was due to carboxylate groups, and the peak for C-H contained in the polysaccharide rings of cellulose was found at 1382 cm^−1^ [68,69]. When H_2_SO_4_ was considered for the further hydrolysis of IL-treated pulp, CNCs were obtained for the C-6 attack of cellulose pyranose ring, as less steric interruption occurs at C-6 than at other carbon ring locations. This can be confirmed by the evidence of the 1230 cm^−1^ peak obtained with increasing acid concentration due to the presence of the sulfate group [70]. Lastly, the evident bands at 1490 and 895 cm^−1^ indicated a pointwise accumulation of calcium oxalate [71].

XRD: The XRD spectrum of CNC_IL_ (Figure 3f) was similar to the one reported in Figure 2d for CNC_AH_. Essentially, the three dominant peaks at 13°, 30°, and 42° can be associated with the crystalline structure of cellulose type II (polymorphic transition from cellulose type I to II). Signals of calcium oxalate could also be detected in CNC_IL_, specifically at 8.3°, 24.2°, and 29°, which are characteristic of this material.

### 3.3. Thermal, Morphological, and Chemical Analysis of Lignin Nanoparticles

One of the most traditional methods of lignin extraction used in the industry is the kraft process, even though it has several major disadvantages, such as high temperature and pressure, the production of environmentally impacting substances, and high water usage. Only recently have authors attempted to prepare nanostructured lignin by mild sonication of kraft liquor [72], thus obtaining spherical LNPs in a sustainable, straightforward, scalable, and organic solvent-free manner. In the past decade, ILs have been used as solvents for natural polymers, including cellulose and starch. This has led to interest in designing and developing ILs that can dissolve or solubilize even lignin and, thus, extract it from lignocellulosic biomass. ILs have been considered for lignin modification and fabrication of advanced materials [73]; recently, Nisha and coworkers recovered lignin from Acacia mangium pulp by treating it with triethylammonium hydrogen sulfate IL [74]. Also, Liu et al. [75] prepared lignin nanospheres from high-concentration lignin–ionic liquid solutions by fragmenting lignin molecules into smaller units with a more homogenous composition and Amin et al. [76] considered the entrapment of LNPs in IL-regenerated cellulose. Nevertheless, studies on the recovery of nanosized lignin from biowaste products using ILs are still few in number [26] and even uncoupled to pulp reuse in a sort of cascading approach. According to this, it is of certain interest to verify if kraft and ILs pulping can have different effects on the characteristics of LNPs recovered from an aquatic species, such as duckweed.

#### 3.3.1. FTIR Analysis of Lignin Nanoparticles from Acid Hydrolysis (LNP_AH_) and Ionic Liquid (LNP_IL_) Treatments

**Figure 4:**
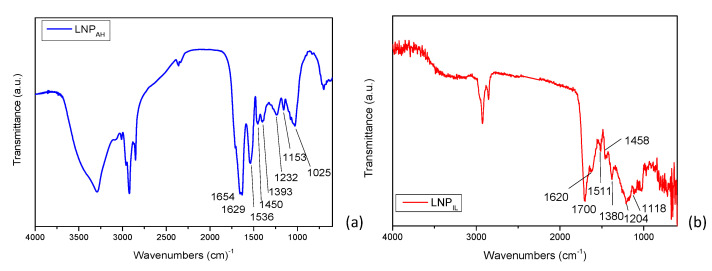
FTIR spectra of LNP_AH_ (**a**) and LNP_IL_ (**b**).

The chemical characterization of the two lignins (LNP_AH_ and LNP_IL_) evidenced specific differences (Figure 4a,b), notable in the fingerprint region. The peak around 1370 cm^−1^ may be attributed to the OH in-plane deformation of alcohols and phenols, and the one at 1205 cm^−1^ to the C-O vibrations of primary alcohols. The C=O stretching around 1700–1660 cm^−1^, aromatic skeletal vibration around 1600 cm^−1^, C-C stretching of the aromatic skeleton around 1540 cm^−1^, C-H stretching of the aromatic skeleton around 1480 cm^−1^, CH vibration of the methyl group at 1440 cm^−1^, syringyl units vibration at 1345 cm^−1^, OH stretching of the primary alcohol at 1040 cm^−1^, and condensed aromatic units at 1120 cm^−1^ can all be assigned to lignin nanoparticles [77]. The absorption band at 1654 cm^−1^ (in the region of a,β-unsaturated carbonyl) indicated that some of the hydroxyl in lignin changed into unsaturated carbonyl during treatment. The IR spectrum of LNP_AH_ also showed a band of 1700 cm^−1^ assigned to the C=O stretching vibrations of hemicelluloses. The not-completely effective removal of polysaccharides for LNP_AH_ is also evident in the appearance of the peak at 897 cm^−1^, corresponding to the pyranose ring opening, and the peaks around 1380 cm^−1^, due to the C-H bending deformation of the polysaccharide band of hemicellulose and 1318 cm^−1^, related to the CH_2_-wagging vibrations in hemicelluloses.

As for [TEA][HSO_4_], Brandt-Talbot et al. [56] reported that products that can be formed during fiber disintegration by the action of this IL are found in solutions with lignin, pseudolignin, and other sugars. The subsequent treatment with ethanol leads to the removal of hemicellulose, while lignin is precipitated by diluting the IL with water and lowering the pH. All these steps determine the effectiveness of the IL-based treatment, and as we found, the lignin obtained from duckweed did not show carbohydrates compared with the lignin obtained via the other method applied in this research.

#### 3.3.2. Morphological and Thermal Characterization of Lignin Nanoparticles from Acid Hydrolysis (LNP_AH_) and Ionic Liquid (LNP_IL_) Treatments

**Figure 5:**
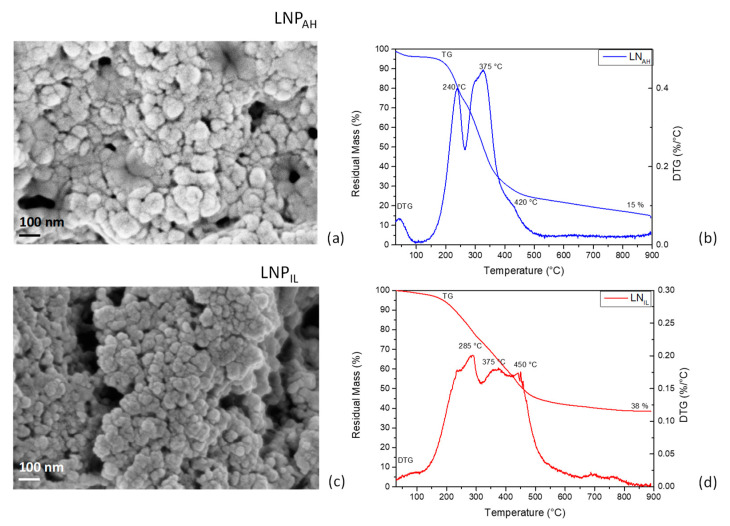
FESEM images of LNP_AH_ (**a**) and LNP_IL_ (**c**); TG*/*DTG curves of LNP_AH_ (**b**) and LNP_IL_ (**d**).

Morphology: Figure 5a,c show FESEM images of cluster-structured lignin nanoparticles with higher mean diameters for LNP_AH_ (82 ± 20 nm) compared with LNP_IL_ (40 ± 11 nm). As indicated above, the precipitation of nanoscaled lignin is achieved by adding water to lignin solubilized by IL [73], as lignin tends to minimize the contact with water to reduce the entropy losses. This allows lignin to organize its highly hydrophobic regions to form a two-phase layer between water and IL, thus entrapping water molecules and creating a balance between the continuous and dispersed phases. Finally, the interactions between the hydrophobic lignin regions lead to small and stable nanoparticles [78]. This process then affects particle growth, giving rise to smaller dimensions when compared with LNPs from acid treatment (LNP_AH_) [79]. Stanisz et al. [80] also found that lignin-based spherical particles can be prepared with the use of hydrogen sulfate ionic liquid; moreover, changes in the morphology of lignin materials can be strictly related to the amount of added ionic liquid, which may also influence the porous structure of the obtained nanoparticles [80].

TGA: The thermal decomposition of lignin nanoparticles can be separated into three major states [81]. In stage I (<150 °C), a small weight loss was observed because of the removal of moisture and volatiles and, eventually, the remaining small amounts of IL. The initial decomposition of lignin is found between 120 and 300 °C (stage II), resulting in fragmentation in the phenyl propane side chains at the end positions, which forms formic acid, formaldehyde, water, carbon dioxide, and sulfur dioxide. The main thermal decomposition occurs between 300 and 500 °C (stage III), with more than 50% of the mass loss related to the release of large MW phenols and gaseous products mainly consisting of CO, CO_2_, and CH_4_. The cracking of the methoxy group and release of methane occurred above 500 °C. In both cases, LNP_AH_ (Figure 5b) and LNP_IL_ (Figure 5d), a shoulder peak was observed, indicating that low-molecular-weight phenolic compounds decompose at lower temperatures and confirming the fragmentation of β-O-4 aryl ether bond of lignin during both treatments, as described by Zou et al. [82]. The intensities of the two sharp DTG peaks were comparable in the case of LNP_AH_, while LNP_IL_ was more difficult to volatilize, as a broad DTG curve with a peak at 375 °C and a left shoulder at 285 °C was observed. The better thermal stability of LNP_IL_ compared with LNP_AH_ was also evident in the value of residual mass measured at the end of the test (38% and 15%, respectively), confirming that lower thermal stability resulted from higher fragmentation of lignin during the pretreatment process. Furthermore, as evidenced by FTIR analysis, the partial removal of hemicellulose was achieved in the case of LNP_AH_, as the peak centered at 240 °C can be related to the degradation of polysaccharide fraction [83], which is also responsible for the higher measured weight loss.

## 4. Conclusions

With an eye on the valorization of natural resources from the perspective of circularity, this research addressed exploiting spontaneous natural resources to obtain nanobiomaterials, which have attracted increasing interest in recent years. In fact, nanostructured biopolymers’ chemical–physical, electronic, and optical properties are the subject of multifaceted technological applications. With this in mind, the present work has, for the first time, explored the valorization of a non-food invasive plant belonging to the freshwater aquatic world, with the aim of obtaining CNC and LNP. To this end, two different biomass processing methods were applied to de-structure the fibers and obtain cellulose and lignin in a nanoscale form. The results showed that materials with different purities, morphologies, sizes, and thermal resistances can be obtained depending on the treatment. In summary, this work offers a new vision and opportunity to obtain nanostructured materials from a freshwater plant, a resource readily available in nature, by modulating the structure and chemical and physical properties of the nanoparticles obtained.

## Data Availability

Dataset available on request from the authors due to privacy.
